# Different controls on the Hg spikes linked the two pulses of the Late Ordovician mass extinction in South China

**DOI:** 10.1038/s41598-022-08941-3

**Published:** 2022-03-25

**Authors:** Zhen Qiu, Hengye Wei, Li Tian, Jacopo Dal Corso, Jiaqiang Zhang, Caineng Zou

**Affiliations:** 1 Research Institute of Petroleum Exploration & Development, China National Petroleum Corporation, Beijing, 100083 China; 2 National Energy Shale Gas Research & Development (Experiment) Center, Beijing, 100083 China; 3 School of Geosciences and Technology, Southwest Petroleum University, Chengdu, 610500 China; 4 State Key Laboratory of Oil and Gas Reservoir Geology and Exploitation (Southwest Petroleum University), Chengdu, 610050 China; 5 State Key Laboratory of Biogeology and Environmental Geology, China University of Geosciences, Wuhan, 430074 China; 6 Institute of Geology and Geophysics, Chinese Academy of Sciences, Beijing, 100029 China

**Keywords:** Solid Earth sciences, Geochemistry, Geology, Tectonics

## Abstract

The Late Ordovician mass extinction (LOME, ca. 445 Ma; Hirnantian stage) is the second most severe biological crisis of the entire Phanerozoic. The LOME has been subdivided into two pulses (intervals), at the beginning and the ending of the Hirnantian glaciation, the LOMEI-1 and LOMEI-2, respectively. Although most studies suggest a rapid cooling and/or oceanic euxinia as major causes for this mass extinction, the driver of these environmental changes is still debated. As other Phanerozoic’s mass extinctions, extensive volcanism may have been the potential trigger of the Hirnantian glaciation. Indirect evidence of intense volcanism comes from Hg geochemistry: peaks of Hg concentrations have been found before and during the LOME, and have all been attributed to global volcanism in origin. Here, we present high-resolution mercury (Hg) profiles in three study sections, from a shelf to slope transect, on the Yangtze Shelf Sea (South China) to address the origin of Hg anomalies across the Ordovician–Silurian (O–S) boundary. The results show Hg anomaly enrichments in the middle Katian, late Katian, the LOMEI-1 at the beginning of the Hirnantian glaciation, the LOMEI-2 in the late Hirnantian glaciation, and late Rhuddanian. The Hg anomaly enrichments during the middle–late Katian and late Rhuddanian would probably reflect a volcanic origin. We find two different controls on the recorded Hg anomalies during the extinction time: i.e., primarily volcanism for the Hg anomaly at the LOMEI-1 and euxinia for the Hg anomaly at the LOMEI-2. Expansion of euxinia at the LOMEI-1 would have been probably enhanced by volcanic fertilization via weathering of volcanic deposits during the Middle and late Katian, and combined with euxinia at the LOMEI-2 to finally be responsible for the two pulses of the LOME.

## Introduction

The Late Ordovician mass extinction (LOME) is the second largest mass extinction of the Phanerozoic^1,2^, and has been linked to the Hirnantian glaciation^2^. The LOME is subdivided into two intervals (LOMEI-1 and LOMEI-2) which occurred at the onset and the end of the Hirnantian glaciation, respectively^2–5^. The LOME is marked by the disappearance of ~ 85% marine species^2,6,7^ or ~ 53% of marine genera^8^. Massive environmental changes were coeval with the LOME, such as rapid global cooling (Hirnantian glaciation) corresponding to expansion of ice sheets on the Gondwana supercontinent^9–12^, and expanded anoxia and/or euxinia in ocean^4,13–17^.

In the geologic record, mass extinction events and global climate changes are often associated to the emplacement of a large igneous province (LIP; e.g., Ref.^18^), but no LIP basalts have been found in the LOME interval, although a Suordakh LIP was postulated^19^ mainly based on the evidence of the poorly aged-constrained Suordakh dolerite eruption within the Katian^20^. Volcanic activity during the LOME has been indirectly inferred by coeval mercury (Hg) peaks found in sedimentary successions (e.g., middle Katian, upper Katian, LOMEI-1 at uppermost Katian and LOMEI-2 in upper Hirnantian) at Wangjiawan (in South China)^21^, Monitor Range (Laurentia)^22^, drillhole XY5 (South China) and Vinini Creek (Laurentia)^23^, and peri-Baltic region^24^.

Sedimentary Hg has been increasingly used as a tracer for volcanic activity during mass extinction events (e.g., ref.^25^). Volcanism is the primary sources of atmosphere Hg before the Anthropocene^26^ and, given its short residence time in the atmosphere, gaseous volcanic Hg is easily transported and deposited in different depositional environments globally^25,27^. Therefore, sedimentary Hg can be used as a proxy for volcanism^25,28–31^. However, increases of Hg deposition in different depositional setting can be also linked to other (local) factors, such as increase of riverine Hg transport to marine settings^32^, massive oxidation of terrestrial organic matter^16,31^, and development of euxinic conditions^33^. Hence, a peak of Hg concentration in the sedimentary record does not unequivocally indicate massive coeval volcanic activity.

In this study, we present new Hg concentration data across the Ordovician–Silurian boundary from three sections at Shuanghe, Qiliao and Tianba on the Yangtze Shelf Sea in South China^4,14^. Combined with previous data on redox conditions and climate changes from the same outcropped sections^4^, these Hg data are used to explore Hg deposition during the LOME in the study area, shedding new light on the origin of the Hg peaks coeval to the biological and environmental changes.

## Geological setting

**Figure 1:**
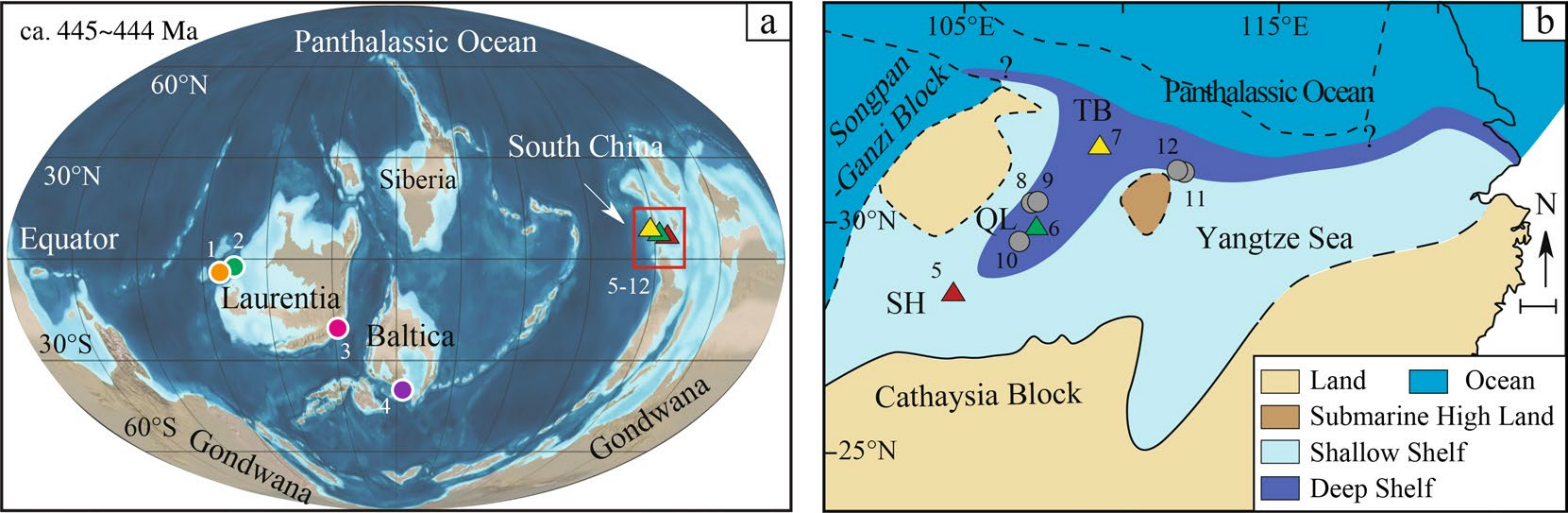
Geological setting. (**a**) Late Ordovician (ca. 445~444 Ma) paleogeography. Adapted from Ron Blakey, © 2016 Colorado Plateau Geosystems Inc.; (**b**) Simplified paleogeographic map of the Yangtze Shelf Sea during the Late Ordovician^4^, showing section localities analysed during the present study, including the Shuanghe (SH) inner shelf outcrop section, Qiliao (QL) mid-shelf outcrop section and the Tianba (TB) outer shelf-slope drill core section. Scale bar = 100 km. Also included in (**a**) and (**b**) are additional sites with Hg anomalies > 100 ppb. These sites comprise: 1. Vinini Creek, United States^23^; 2. Monitor Range, United States^22^; 3. Dob's Linn, Scotland^53^; 4. Zbrza PIG-1, Poland^24^; 5. Shuanghe (SH), South China; 6. Qiliao (QL), South China; 7. Tianba (TB), South China; 8. Yanzhi, South China^48^; 9. XY5, South China^23^; 10. Jiaoye, South China^48^; 11. Wangjiawan, South China^21,22^; 12. Dingjiapo, South China^21^.

**Figure 2:**
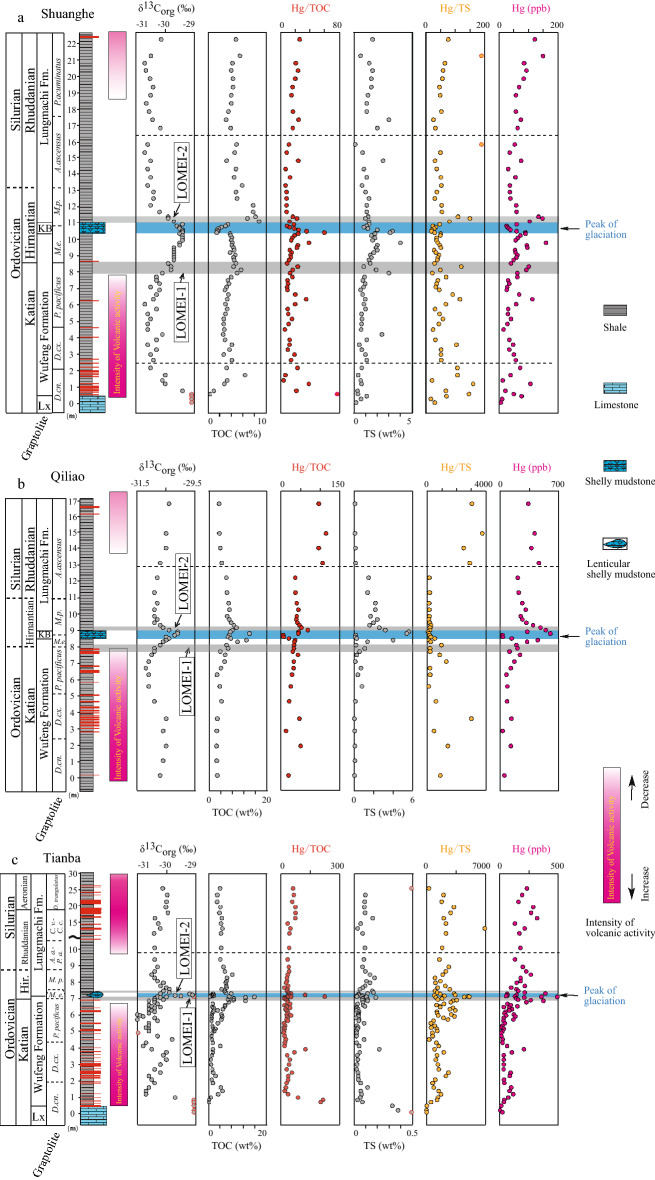
Chemostratigraphy of organic-carbon isotope, Hg/TOC, TOC, Hg/TS, TS, Hg concentration and Fe species across the O–S boundary from the Shuanghe (**a**), Qiliao (**b**) and Tianba (**c**) in South China. Organic-carbon isotope and Fe species data are from Ref.^4^. TOC data are mainly from Ref.^4,14^. Intensity of volcanic activity was estimated by distribution and thickness of volcanic ash layers deposited in South China across the Ordovician and Silurian transition. Graptolite zones: *D. cn.* *Dicellograptus complanatus*; D. cx. *Dicellograptus complexus*; *P. pacificus* *Parakidograptus pacificus*; *M. e.* *Metabolograptus extraodinarius*; *M. P.* *Metabolograptus persculptus*; *A. a.* *Akidograptus ascensus*; *P. a.* *Parakidograptus acuminatus*; *C. v.* *Cystograptus vesiculosus*; *C. c.* *Coronograptus cyphus*.

During the Ordovician–Silurian transition, the Yangtze Platform (South China Block) was located near the equator^34^ (Fig. 1). After the middle Katian, it gradually evolved into a siliciclastic-dominated shelf basin, called the Yangtze Shelf Sea^4,35^. The shale strata, which include the Late Ordovician Wufeng Formation and early Silurian Lungmachi Formation, or Wufeng-Lungmachi Shale, deposited on the shelf with deepening northwards to the Panthalassic Ocean^4,36,37^. The bottom black shale interval of Wufeng-Lungmachi Shale in South China corresponds to typical, organic-rich shales (hot shale, i.e., more radioactive shale)^7,38^. Owing to the glaciation, a rapid sea level drop occurred during the Hirnantian. Paleo-water-depth in the Yangtze Shelf Sea during this glaciation was likely about 40–100 meters^37^. The Kuanyinchiao Bed at the top of the Wufeng Formation was formed during this glaciation time (Fig. 2), and contains characteristic cold water Hirnantian fauna. The marine carbonate Kuanyinchiao Bed is widely distributed, and has a conformity contact with the underlying Wufeng Formation in the study area. High-resolution graptolite zones have been previously identified in South China^39^, providing a solid biostratigraphic framework and allowing correlation with other Ordovician–Silurian boundary sections around the world^7^.

From the Late Ordovician to early Silurian, volcanic ash layers deposited are extensively reported, especially in North America^40^ and South China^41–43^. In North America, over 100 volcanic ash layers dominantly occurred in pre-late Katian stage^40^. There were two pulses of volcanic ash layers deposited in South China in the Late Ordovician and early Silurian^42^. The first pulse occurred in late Katian stage^41,42,44^ and the second pulse erupted around the boundary between the Rhuddanian stage and the Aeronian stage (ca. 440.8 Ma)^42,45^.

## Materials and methods

Fresh rock samples were collected from three sections (Shuanghe, Qiliao and Tianba sections) that were deposited from proximal to distal areas on the Yangtze Shelf Sea, South China (Fig. 1). For each section, high-resolution graptolite zones have been previously defined^4^. Previous studies have reported, from the same rock samples, TOC contents, C-isotopes, Fe-speciation, major elements and trace elements concentrations^4,12,46^. In this study, we measured Hg concentration and TS content of all samples, and the TOC content of 6 new samples.

Hg concentration was measured using a Lumex RA-915 M mercury analyzer with pyrolyzer PYRO-915 + at State Key Laboratory of Biogeology and Envionmental Geology, China University of Geosciences. An aliquot of ~ 50 mg of powdered sample was weighed in a glass boat and was heated in the pyrolyzer at 700 °C. Volatilized Hg concentration was quantified via atomic absorption spectrometry. A soil standard (GSD-17a; Hg = 120 ± 10) was used to calibrate the instrument. Repeated measurements of the standard at the start of each run and throughout the analysis sequence indicate reproducibility was generally better than 10% for Hg concentrations.

For the measurement of TOC content, sample powders (2 g) were decarbonated with HCl (10% vol/vol) prior to TOC analyses on a LECO CS-230 analyzer. TS was measured directly by bulk sample. Analytical precision was generally better than 5% and 8% for TOC, and TS contents, respectively. Hg concentrations have been normalized for TOC and TS contents^25,33^.

## Results

At Shuanghe section, Hg concentrations range from 5 to 161 ppb (average is 61 ppb) (Table 1). Background Hg concentration is ~ 50 ppb. Higher Hg concentrations are found at the base of the lower Katian Wufeng Formation, in the upper Katian, LOMEI-1 (end-Katian), LOMEI-2 (Hirnantian), and in the upper Rhuddanian (Fig. 2). At Qiliao section, Hg concentrations range from 27 to 600 ppb (average is 245 ppb) (Table 1). Background Hg concentration is ~ 80 ppb. Higher Hg concentrations occur at LOMEI-1, LOMEI-2, and in the upper Rhuddanian (Fig. 2). At Tianba section, Hg concentrations range from 8 to 498 ppb (average is 130 ppb) (Table 1). Background Hg concentration is ~ 45 ppb. Hg spikes occur in the upper Katian, LOMEI-1, LOMEI-2, and upper Rhuddanian (Fig. 2).

**Table 1:** Hg concentration, TOC and TS contents, Hg/TOC, Hg/TS ratios at Shuanghe (SH), Qiliao (QL) and Tianba (TB) sections, South China. ^4,14^

Section	Height	FM	Sample number	δ^13^C_org_^#^	TOC^#,^*	Hg	Hg/TOC	TS	Hg/TS
	(m)			(‰)	(wt%)	ppb	ppb/%C	(wt%)	ppb/%S
SH	22.19	LM	C225	− 30.1	4.8	122	25	1.6	76
SH	21.19	LM	C224	− 30.5	5.5	151	27	0.6	265
SH	20.74	LM	C001	− 30.7	4.2	85	20	1.3	65
SH	20.29	LM	C222	− 30.7	4.0	93	23	1.6	58
SH	19.79	LM	C221	− 30.5	4.3	84	20	1.5	56
SH	19.29	LM	C220	− 30.4	4.3	69	16	1.5	46
SH	18.79	LM	C219	− 30.5	4.0	58	14	1.2	48
SH	18.29	LM	C218	− 30.7	4.0			1.1	
SH	17.79	LM	C217	− 30.6	3.5	58	16	1.1	52
SH	17.29	LM	C216	− 30.4	3.1	75	24	3.0	25
SH	16.79	LM	C215	− 30.2	3.9	64	16	2.0	32
SH	15.79	LM	C214	− 30.6	4.9	53	11	0.1	485
SH	15.29	LM	C002	− 30.7	4.6	36	8	0.7	49
SH	14.79	LM	C212	− 30.5	3.2	76	24	2.5	30
SH	14.29	LM	C211	− 30.6	4.8	31	6	0.9	36
SH	13.79	LM	C210	− 30.5	4.5	56	12	1.1	51
SH	13.3	LM	C209	− 30.6	5.9	37	6	0.7	52
SH	12.9	LM	C208	− 30.4	4.8	37	8	1.0	37
SH	12.5	LM	C207	− 30.4	5.0	38	8	1.3	30
SH	12.08	LM	C206	− 30.5	7.8	59	8	1.1	53
SH	11.68	LM	C205	− 30.2	7.8	60	8	1.1	54
SH	11.39	LM	C003	− 29.9	8.2	135	16	1.2	110
SH	11.29	LM	C203	− 29.9	6.8	149	22	1.0	151
SH	11.09	LM	C201	− 29.7	8.8	104	12	1.3	80
SH	10.89	KB	C199	− 29.4	3.4	60	18	1.3	46
SH	10.79	KB	C004	− 29.6	3.0	25	8	1.1	23
SH	10.71	KB	C197	− 29.5	2.3	28	12	0.9	33
SH	10.63	KB	C196	− 29.5	1.8	33	18	1.2	28
SH	10.55	KB	C005	− 29.4	1.6	38	24	2.0	19
SH	10.47	KB	C194	− 29.4	1.7	60	35	3.3	18
SH	10.38	KB	C193	− 29.4	1.5	90	60	3.1	29
SH	10.32	WF	C191	− 29.5	3.8	90	24	1.8	50
SH	10.22	WF	C190	− 29.4	3.9	73	19	1.5	49
SH	10.07	WF	C006	− 29.4	4.1	64	16	1.8	36
SH	9.91	WF	C188	− 29.4	4.0	57	14	1.6	35
SH	9.77	WF	C187	− 29.5	4.2	161	38	4.0	40
SH	9.61	WF	C186	− 29.5	4.1	95	23	2.0	47
SH	9.46	WF	C185	− 29.7	4.4	96	22	1.9	51
SH	9.32	WF	C184	− 29.7	4.5	55	12	2.0	27
SH	9.16	WF	C183	− 29.7	3.8	56	15	1.7	33
SH	9.02	WF	C182	− 29.7	4.7	63	13	1.4	45
SH	8.86	WF	C007	− 29.7	4.4	60	14	1.5	40
SH	8.71	WF	C180	− 29.7	4.5	50	11	1.3	38
SH	8.51	WF	C179	− 29.9	4.8	40	8	1.4	29
SH	8.3	WF	C178	− 29.8	4.4	101	23	0.8	120
SH	8.1	WF	C177	− 29.8	5.7	93	16	1.9	49
SH	7.91	WF	C176	− 30.1	5.0	63	13	3.0	21
SH	7.68	WF	C175	− 30.3	4.0	63	16	0.9	69
SH	7.47	WF	C174	− 30.3	3.4	31	9	0.8	40
SH	7.27	WF	C173	− 30.2	3.6	35	10	1.0	36
SH	7.07	WF	C008	− 30.5	3.2	27	9	0.8	33
SH	6.9	WF	C171	− 30.2	2.9	26	9	0.6	45
SH	6.61	WF	C170	− 30.3	3.5	69	20	0.8	92
SH	6.32	WF	C169	− 30.4	3.3	114	34	1.0	116
SH	6.02	WF	C168	− 30.7	3.0	58	19	0.9	68
SH	5.72	WF	C167	− 30.3	3.3	31	9	1.0	31
SH	5.42	WF	C166	− 30.6	3.0	30	10	0.7	46
SH	5.12	WF	C165	− 30.5	2.7	41	15	0.7	58
SH	4.82	WF	C164	− 30.6	2.7	28	10	0.7	44
SH	4.48	WF	C009	− 30.6	2.8	15	5	0.6	24
SH	4.18	WF	C162	− 30.1	2.6			2.4	
SH	3.85	WF	C161	− 30.3	2.9	35	12	1.1	32
SH	3.55	WF	C160	− 30.6	4.1	48	12	0.5	104
SH	3.25	WF	C159	− 30.3	3.7	35	9	0.7	51
SH	2.95	WF	C158	− 30.5	2.7	51	19	1.0	51
SH	2.6	WF	C157	− 30.4	4.1	57	14	1.1	52
SH	2.15	WF	C156	− 30.5	3.2	72	23	0.7	108
SH	1.7	WF	C155	− 30.0	6.4	39	6	0.4	108
SH	1.38	WF	C010	− 30.1	3.3	12	4	0.6	20
SH	1.16	WF	C153	− 30.0	2.8	107	38	0.7	162
SH	0.74	WF	C150	− 29.4	1.0	21	21	0.3	68
SH	0.54	WF	C149	− 26.1	0.3	50	167	0.3	147
SH	0.41	LX	C148	− 27.8	0.1	77	770	1.1	70
SH	0.24	LX	C147	− 28.1	0.1	10	97	0.6	16
SH	0	LX	C146	− 27.7	0.1	5	53	0.2	29
QL	16.69	LM	C340	− 30.4	3.5	336	96	0.1	3054
QL	14.89	LM	C336	− 30.5	3.6	413	115	0.1	3751
QL	13.99	LM	C333	− 30.5	3.9	374	96	0.2	2492
QL	13.09	LM	C330	− 30.7	4.4	465	106	0.2	2906
QL	12.19	LM	C327	− 30.8	6	209	35	1.5	139
QL	11.29	LM	C324	− 30.9	6.2	220	35	1.4	157
QL	10.65	LM	C321	− 30.8	6.8	263	39	2.2	120
QL	10.25	LM	C319	− 30.9	7.1	305	43	2.2	139
QL	9.85	LM	C317	− 30.9	6.3	244	39	1.7	144
QL	9.65	LM	C316	− 30.8	7	265	38	2.4	110
QL	9.45	LM	C315	− 30.9	7.7	313	41	2.0	156
QL	9.3	LM	C314	− 30.7	8.6	394	46	1.6	246
QL	9.15	LM	C313	− 30.6	9.5	485	51	2.6	186
QL	9	LM	C312	− 30.4	8.1	547	68	3.2	171
QL	8.9	KB	C311	− 30.1	7.4	324	44	5.6	58
QL	8.8	KB	C310	− 30.1	14	600	43	5.4	111
QL	8.7	KB	C309	− 30.2	7	27	4	0.2	156
QL	8.6	KB	C308	− 30.5	6.6	31	5	0.1	240
QL	8.5	KB	C307	− 30.4	7.5	136	18	0.3	503
QL	8.4	WF	C306	− 30.5	13	451	35	4.0	113
QL	8.3	WF	C305	− 30.6	9.9	322	32	1.4	230
QL	8.1	WF	C303	− 30.7	6.1	192	31	0.2	960
QL	7.9	WF	C301	− 30.9	6.6	205	31	1.1	187
QL	7.7	WF	C299	− 30.9	3.9	111	28	0.6	191
QL	7.5	WF	C297	− 31	5.6	236	42	0.3	841
QL	7.09	WF	C295	− 31.1	5.4	168	31	0.1	1291
QL	6.69	WF	C293	− 31.2	4.1	126	31	0.7	177
QL	6.29	WF	C291	− 31.1	2.8	74	26	0.4	190
QL	5.54	WF	C288	− 31.1	3.7	85	23	0.8	104
QL	4.64	WF	C285	− 30.9	4.3	81	19	0.1	581
QL	3.58	WF	C282	− 30.5	2.9	133	46	0.0	3011
QL	2.83	WF	C279	− 30.6	2.7	29	11	0.1	449
QL	1.92	WF	C276	− 30.5	2.6	127	49	0.1	1400
QL	1.02	WF	C273	− 30.6	2.7			0.1	
QL	0.12	WF	C270	− 30.7	2.9	50	17	0.1	877
TB	25.36	LM	C107	− 30.1	4.0	235	59	0.9	255
TB	23.36	LM	C106	− 30.0	3.1	193	62	0.1	1991
TB	21.36	LM	C105	− 30.0	3.0	161	54	0.1	1774
TB	19.56	LM	C104	− 30.0	4.1	289	70	0.1	3245
TB	17.81	LM	C103	− 30.0	3.8	271	71	0.1	2849
TB	16.21	LM	C102	− 30.4	4.6	324	70	0.2	2023
TB	14.58	LM	C101	− 30.3	4.3	130	30	0.1	2286
TB	13.08	LM	C100	− 30.2	4.8	215	45	0.0	6932
TB	11.58	LM	C099	− 30.4	4.3	170	39	0.1	2291
TB	10.08	LM	C098	− 30.4	4.5	182	40	0.1	1872
TB	9.38	LM	C097	− 30.4	4.2	134	32	0.1	1538
TB	8.92	LM	C096	− 30.3	5.3	203	38	0.1	2664
TB	8.64	LM	C095	− 30.4	5.8	231	40	0.1	2312
TB	8.44	LM	C094	− 30.5	8.4	272	32	0.1	1939
TB	8.24	LM	C093	− 30.4	6.5	184	28	0.2	1019
TB	8.04	LM	C092	− 30.3	6.4	183	29	0.1	1665
TB	7.94	LM	C091	− 30.1	6.1	149	24	0.1	1063
TB	7.74	LM	C090	− 30.2	6.3	220	35	0.1	2240
TB	7.64	LM	C089	− 30	6.4	102	16	0.1	1326
TB	7.54	LM	C088	− 29.9	4.7	148	31	0.1	2787
TB	7.44	LM	C087	− 30.1	5.7	163	29	0.2	1016
TB	7.34	LM	C086	− 29.9	6.1	218	36	0.2	1285
TB	7.28	LM	C085	− 30.2	8.0	398	50	0.1	3314
TB	7.25	KB	C084	− 29.2	1.8	69	38	0.0	1638
TB	7.19	KB	C082	− 29.1	1.2	148	123	0.0	3442
TB	7.16	KB	C081	− 29.7	2.0	69	35	0.1	936
TB	7.13	KB	C080	− 29.5	1.5	60	40	0.0	2139
TB	7.1	KB	C079	− 28.6	0.6	135	224	0.0	4985
TB	7.09	WF	C078	− 30.3	7.3	363	50	0.1	5118
TB	7.07	WF	C077	− 30	16.0	498	31	0.1	4526
TB	7.04	WF	C076	− 30.2	13.0	215	17	0.2	1129
TB	6.98	WF	C074	− 30.4	8.9	145	16	0.1	1863
TB	6.92	WF	C256	− 30.1	6.9	162	23	0.1	1346
TB	6.88	WF	C072	− 30.4	8.8	271	31	0.1	3611
TB	6.84	WF	C071	− 30.6	13.0	380	29	0.1	3170
TB	6.76	WF	C069	− 30.4	7.2	233	32	0.1	2915
TB	6.66	WF	C067	− 30.3	1.6	71	45	0.0	2094
TB	6.58	WF	C065	− 30.6	5.9	98	17	0.1	1198
TB	6.51	WF	C064	− 30.5	5.8	155	27	0.1	1617
TB	6.46	WF	C063	− 30.2	1.3	39	30	0.0	1642
TB	6.41	WF	C062	− 30.4	1.9	86	45	0.0	3167
TB	6.33	WF	C061	− 30.6	5.1	109	21	0.0	2803
TB	6.25	WF	C059	− 30.5	3.3	86	26	0.0	3448
TB	6.17	WF	C058	− 30.4	1.8	56	31	0.0	1242
TB	6.08	WF	C057	− 30.6	1.3	46	35	0.0	1269
TB	6	WF	C056	− 31	4.6	125	27	0.0	2969
TB	5.94	WF	C055	− 30.9	1.2	60	50	0.0	1393
TB	5.88	WF	C054	− 30.8	4.8	138	29	0.0	3361
TB	5.79	WF	C053	− 30.4	1.2	39	32	0.0	2042
TB	5.69	WF	C052	− 31	4.6	121	26	0.0	2572
TB	5.61	WF	C051	− 30.3	1.1	37	33	0.0	832
TB	5.46	WF	C049	− 30.6	3.4	49	14	0.1	541
TB	5.3	WF	C048	− 30.6	1.4	27	19	0.0	981
TB	5.19	WF	C047	− 30.7	1.6	22	14	0.0	567
TB	5.11	WF	C046	− 30.6	2.5	39	16	0.0	1225
TB	4.98	WF	C044	− 30.6	2.2	29	13	0.0	1278
TB	4.87	WF	C043	− 31.1	1.4	18	13	0.0	720
TB	4.67	WF	C042	− 30.3	1.1	19	17	0.0	744
TB	4.47	WF	C041	− 29.8	1.3	30	23	0.1	329
TB	4.27	WF	C040	− 30.7	1.6	29	18	0.0	1168
TB	4.07	WF	C039	− 30.8	4.2	68	16	0.0	1455
TB	3.87	WF	C143	− 30.5	1.7	212	125	0.2	964
TB	3.68	WF	C038	− 30	1.6	108	67	0.1	2069
TB	3.5	WF	C037	− 30.1	1.3	39	30	0.0	1261
TB	3.23	WF	C036	− 30	0.9	39	43	0.0	1114
TB	2.93	WF	C035	− 30.2	1.0	39	39	0.0	1355
TB	2.63	WF	C033	− 30.4	1.4	38	27	0.0	1462
TB	2.43	WF	C032	− 30.2	1.8	98	54	0.0	2638
TB	2.22	WF	C031	− 30.6	2.6	91	35	0.0	2328
TB	2	WF	C030	− 30.5	1.2	42	35	0.0	1040
TB	1.8	WF	C029	− 30.4	1.7	44	26	0.1	740
TB	1.53	WF	C028	− 30.6	4.4	103	23	0.1	855
TB	1.31	WF	C027	− 30.7	5.0	89	18	0.1	1459
TB	1.11	WF	C026	− 30.7	3.5	120	34	0.1	1735
TB	0.91	WF	C025	− 29.7	0.9	78	86	0.1	1360
TB	0.76	WF	C024	− 27.1	0.2	43	215	0.0	1265
TB	0.63	WF	C023	− 27.6	0.2	41	203	0.1	541
TB	0.4	LX	C022	− 28.2	0.1	18	181	0.3	55
TB	0.12	LX	C021	− 27.2	0.1	8		0.4	22
TB	0	LX	C020	− 27.6	0.1	27	273	0.9	32

*FM* formation, *LM* Lungmachi, *KB* Kuangyinchiao bed, *WF* Wufeng.

*TOC data for samples C102-C107 were analyzed for the present study.

^#^δ13Corg data and most of TOC data from Refs. ^4,14,46^.

At Shuanghe, TOC contents range from 0.1 to 8.8% (average is 3.8%)^4,14^ (Table 1). Higher TOC values occur at base of the early Katian Wufeng Formation, LOMEI-1, and LOMEI-2 (Fig. 2). At Qiliao, TOC contents range from 2.6 to 14% (average is 6.0%)^4^ (Table 1). Higher TOC values occur at LOMEI-1 and LOMEI-2 (Fig. 2). At Tianba, TOC contents range from 0.1 to 16% (average is 3.8%) (Table 1). TOC increases at base of the lower Katian Wufeng Formation, LOMEI-1, and LOMEI-2, respectively (Fig. 2).

At Shuanghe, TS contents range from 0.1 to 4.0% (average is 1.3%) (Table 1). TS increases at base of the low-Katian Wufeng Formation, in the upper Katian, and at LOMEI-1 and LOMEI-2 (Fig. 2). At Qiliao, TS contents range from 0.1 to 5.6% (average is 1.3%) (Table 1). Higher TS occurs at LOME-1 and LOME-2 (Fig. 2). At Tianba, TS contents range from 0.1 to 0.5% (average is 0.16%) (Table 1). TS is higher in lower Katian Linxiang Formation, and at LOMEI-1 and LOMEI-2 (Fig. 2).

At Shuanghe, peaks of Hg/TOC occur in the middle Katian at the base of the Wufeng Formation (from ~ 20 to ~ 80 ppb/wt.%), end-Katian LOMEI-1 (from ~ 10 to 30 ppb/wt.%) and late-Hirnantian LOMEI-2 horizons (from ~ 18 to ~ 60 ppb/wt.%) (Fig. 2). At Qiliao, peaks of Hg/TOC occur at LOMEI-1 (~ 20 to ~ 50 ppb/wt.%), LOMEI-2 (from ~ 40 to ~ 65 ppb/wt.%), and in the upper Rhuddanian (from ~ 50 to ~ 100 ppb/wt.%) (Fig. 2). At Tianba, peaks of Hg/TOC occur in the middle-Katian Linxiang Formation (from ~ 30 to ~ 280 ppb/wt.%), in the upper Katian (from ~ 30 to ~ 140 ppb/wt.%), and at LOMEI-1 (from ~ 40 to ~ 220 ppb/wt.%) (Fig. 2).

At Shuanghe, peaks of Hg/TS occur at the middle-Katian Linxiang Formation (from ~ 40 to ~ 150 ppb/wt.%), upper Katian, LOMEI-1 (from ~ 40 to ~ 120 ppb/wt.%) and LOMEI-2 horizons (from ~ 40 to ~ 150 ppb/wt.%) (Fig. 2). At Qiliao, peaks of Hg/TS occur in upper Katian (from ~ 1000 to ~ 3000 ppb/wt.%), at the LOMEI-1 (from ~ 250 to ~ 1200 ppb/wt.%), and in the upper Rhuddanian (from ~ 200 to ~ 3200 ppb/wt.%). At Tianba, peaks of Hg/TS occur in the upper Katian (from ~ 70 to ~ 2400 ppb/wt.%), LOMEI-1 horizon (from ~ 700 to ~ 5200 ppb/wt.%), LOMEI-2 horizon (from ~ 1700 to ~ 3500 ppb/wt.%), and in the upper Rhuddanian (from ~ 1800 to ~ 7000 ppb/wt.%) (Fig. 2).

## Discussion

### Hg host and digenesis

Hg concentrations in sediments are controlled mainly by local depositional environment, primary volcanic loading, and post depositional diagenesis^47^. In general, Hg is mainly associated with organic matter under “normal” conditions and with sulfide only under strong euxinic conditions^28,47,48^. Hg/TOC or Hg/TS ratios are then used to assess the excess input of Hg besides of the sources from Hg-TOC complexes or HgS^33,48,49^.

**Figure 3:**
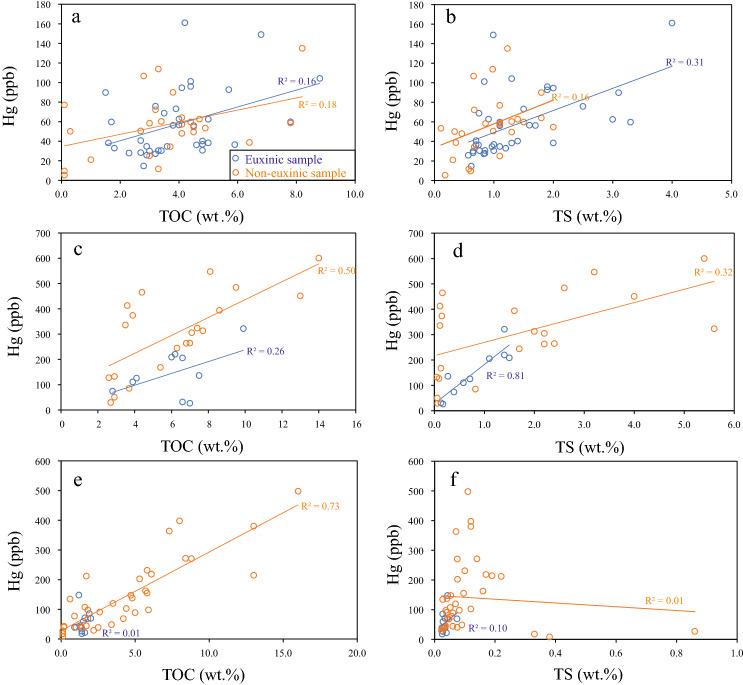
Crossplots of TOC vs. Hg, TS vs. Hg of samples from euxinic and non-euxinic conditions across the Ordovician–Silurian boundary at Shuanghe (**a**,**b**), Qiliao (**c**,**d**), and Tianba (**e**,**f**). The euxinic and non-euxinic conditions are determined by Fe species and trace elements from ref.^4^.

Cross-plots between TOC and Hg (Fig. 3) show that Hg concentrations have no correlation with TOC contents under euxinic environments but have positive correlation (R^2^ is 0.5 to 0.73, Qiaoliao and Tianba sections) or weak correlation (R^2^ = 0.18, Shuanghe section) with TOC contents under non-euxinic environments. It suggests that organic matter is an important host of Hg in these marine sedimentary rocks owing to the association between Hg and organic matter and the reactive Hg organic complexes^50^. Cross-plots between TS and Hg (Fig. 3) show strong correlation (R^2^ = 0.81, Qiaoliao section) to no correlation (R^2^ is less than 0.31, Shuanghe and Tianba sections) under euxinic environments, but show no correlation under non-euxinic environments. This suggests that sulfide is not a host of Hg under non-euxinic conditions but may be a host of Hg during euxinic intervals^48^.

Normalization of Hg concentrations to TOC and TS can reflect Hg anomalies. Hg/TOC ratios could be inflated because of diagenetic degradation of organic matter, which lowers TOC content^22^, or due to analytical uncertainty for TOC < 0.2%^51^. Therefore, high Hg/TOC ratios in the upper Linxiang Formation of the middle Katian at Shuanghe and Tianba (Table 1) do not indicate true positive Hg anomalies, but are linked to very low TOC values (< 0.1%), and are not plotted in Fig. 2. However, the high Hg/TOC ratios in the lowermost Wufeng Formation are associated with TOC contents higher than 0.2%, suggesting true Hg positive anomalies in the middle Katian at Shaunghe and Tianba. In addition, all other recorded high Hg/TOC ratios are associated with TOC > 2.0%^22^.

A diagenetic effect is excluded because of the well-preserved conditions of primary laminated shale^4,14^. In addition, organic-carbon isotope data record the global positive excursion of the glacial interval in the Hirnantian stage, also suggesting a primary chemostratigraphic signal preserved in the sample containing matured organic matter^52^. These analyses point to no or weak diagenetic effect on our geochemical data across the O–S boundary.

### Hg enrichment pattern across the Ordovician–Silurian transition

**Figure 4:**
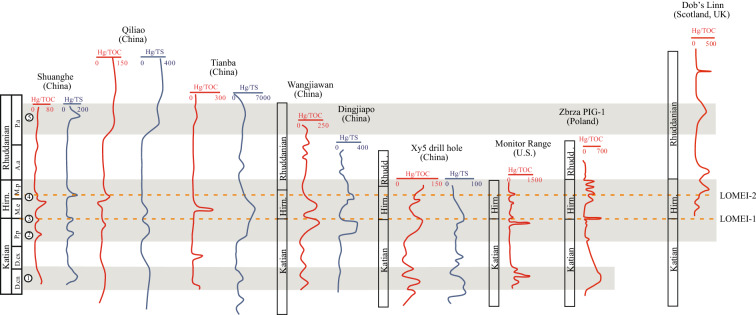
Chemostratigraphic correlation of Hg anomalies across the Ordovician–Silurian boundary. The abbreviation for graptolite zone can be seen in Fig. 2. ① to ⑤: Hg anomalies; Data of Wangjiawan and Dingjiapo in South China from Ref.^21^; Data of XY5 in South China from Ref.^23^; Data of Monitor Range in United States from Ref.^22^; Data of Zbrza PIG-1in Poland from Ref.^24^; Data of Dob's Linn in Scotland from Ref.^53^.

We correlate the Hg/TOC and Hg/TS curves across the Ordovician–Silurian boundary in the three study sections, with the published Hg curves elsewhere (Fig. 4). These Hg anomalies all correspond to Hg concentration peak and peaks of Hg/TOC and/or Hg/TS ratios, indicating increase of Hg input. Two Hg positive anomalies occur in the middle and late Katian, three anomalies occur at LOMEI-1, LOMEI-2 and Late Rhuddanian, respectively (Fig. 4). Elsewhere, one Hg positive anomaly had also been found in late Katian from a drill hole XY5 in South China^23,52^ and Monitor Range in North America^22^. Two Hg positive anomalies at LOMEI-1 and LOMEI-2 had also been found in many locations such as Wangjiawan, Dingjiapo^21^, XY5 drill hole^52^ in South China and Monitor Range section in North America^22^. In other locations in South China, Hg positive anomalies were found in the early Rhuddanian^48^. Hg positive anomalies in Poland in the middle Katian, LOMEI-1, and LOMEI-2 were reported, respectively^24^. Besides, Hg positive anomalies at LOMEI-2 and in the Rhuddanian were reported in Scotland^53^. Overall, the Hg anomaly in the middle Katian in our study sections can be correlated with those in other location in South China^52^, Monitor Range in U.S.^22^, and in Poland^24^. The Hg anomalies in the upper Katian and at LOMEI-1 in our study section can be correlated with those in other locations in South China^21,52^, Monitor Range in U.S.^22^, and Poland^24^. The Hg positive anomaly at LOMEI-2 in our study sections can be correlated with those in other locations in South China^21,52^, Monitor Range in U.S.^22^, Poland^24^, and Scotland^53^. The Hg positive anomaly in the upper Rhuddanian in our study sections can be correlated with those in Scotland^53^ and other location in South China^48^.

### Origin of the Hg anomalies in the study sections

The positive Hg anomalies (i.e., peaks of Hg/TOC and/or Hg/TS) in the middle and late Katian are associated with relative high TOC and TS contents at Shuanghe and Qiliao sections, indicating that this upper Katian Hg enrichment was not related to higher TOC or sulfide fluxes and reflect an enhanced Hg loading into the basins, supporting their interpretation as genuine Hg positive anomalies^21,22,24,52^. Abundant ash beds in the middle and upper Katian in South China^42–44,54^ (Fig. 2) and several ash beds in middle Katian in Poland^24^ have been found. Considering the regional/global signal of Hg anomaly in the middle and late Katian mentioned above, the inferred volcanism in South China or a ‘LIP’ in the middle-late Katian^20,40,55^ may contribute to the two positive Hg anomalies in the middle and late Katian in this study sections and other locations^21–23^, and large mass-independent sulfur isotope anomalies^52^. In other words, the Hg positive anomalies in the middle and late Katian suggest a volcanic increasing Hg loading during this time.

High Hg/TOC or Hg/TS ratios at the LOMEI-1 at the end-Katian in the three study sections are associated with relatively high TOC or TS contents, and high Hg concentrations, also suggesting higher Hg loading into the basins. The Hg spike at the LOMEI-2 at Shuanghe was associated with high TOC contents, invariable Hg/TOC ratios & TS contents, and high Hg/TS ratios, indicating that this Hg enrichment at Shuanghe was linked to higher TOC fluxes. Meanwhile, the Hg spike at the LOMEI-2 at Qiliao was associated with high TS contents, invariable Hg/TS ratios, relatively high TOC contents and Hg/TOC ratios (Fig. 2), suggesting that Hg enrichment at Qiliao might be related to local sulfide deposition. Sulfide-carrier Hg anomaly was also reported by previous study with coarse resolution across the LOME interval^48^. Hg profile at Tianba does not show any positive anomaly at the LOMEI-2, but the good covariation of Hg-TOC (Fig. 3e) suggests organic matter carrier of Hg during this time.

Overall, Hg positive anomaly at LOMEI-1 record higher loading of Hg into the basin, while an increase of Hg drawdown at LOMEI-2 due to more reducing conditions^4^ or increase of organic matter deposition in the late Hirnantian (Fig. 2). Higher Hg fluxes in the basins at LOMEI-1 could be related to different factors, as an increase of volcanic activity or enhanced continental weathering^56,57^. Considering the relatively low continental weathering indicated by chemical index alteration (CIA) values^4^ and the sporadical occurrence of ash beds in South China^42,46^ (Fig. 2), the abrupt Hg (and Hg/TOC) anomalies at LOMEI-1 would be of volcanic origin as suggested by previous published works in South China and U.S.^21–24^. However, intermittent or weak euxinia also developed during the LOMEI-1^4^ with high Hg/TOC or Hg/TS ratios, relatively high TOC or TS contents in the three study sections, suggesting Hg enrichment during this time would be partially related to euxinic condition in water column.

The relatively high Hg concentration in the upper Rhuddanian is associated with non-euxinic conditions, relatively low TOC or TS contents, but relatively high Hg/TOC and Hg/TS ratios in this study. The abrupt increasing of Hg/TOC, or Hg/TS ratios coincides with invariable or small decreasing TOC or TS, excluding an inflation of too low TS and TOC contents. These probably suggest a genuine Hg positive anomaly and extra environmental loading related to volcanism or weathering^56,57^. This anomaly is associated with abrupt occurrence of frequent ash beds^42^ in Fig. 2 and relatively low weathering of CIA evidence^10^, pointing to a volcanic origin of increasing Hg loading. Previous study^53^ has also suggested that this anomaly in the Scotland derived from increased Hg flux rather than sequestration by anoxia/euxinia. These may indicate global Hg loading by volcanism during the late Rhuddanian.

### Implications for the mass extinction

The interpretation of two different controls on the recorded Hg anomalies, i.e., a mixed origin of volcanism and euxinia for the Hg anomaly at the LOMEI-1 and a redox control on the Hg anomaly at the LOMEI-2, shed new lights on the Late Ordovician extinction mechanism. The middle-Katian Hg positive anomaly was temporally coincided with the middle-Katian genus richness drop^58^. The inferred volcanic-origin (although lack of solid volcanic lithology evidences) of Hg positive anomaly in the middle Katian in this study may suggest significant climatic effect triggered by volcanism on the initial of long-term mass extinction from middle Katian to late Hirnantian^58,59^.

The most distinct Hg anomaly and volcanism reported by previous studies are in the Late Katian and at LOMEI-1 (at end-Katian) (Fig. 4). As shown in Fig. 2, intensity of volcanic activity estimated by distribution and thickness of volcanic ash layers gradually decreased from the middle Katian to end-Katian. However, long-term weathering of volcanic deposits would enhance the nutrient input to ocean and thus result in large amount of organic matter burial and the consumption of CO_2_ in the atmosphere via biological pump^59–61^. Increasing burial rates of organic matter would gradually contribute to expansion of euxinia in bottom water^4^, finally driving the LOMEI-1. Additionally, Hg toxic effect could also have contributed to this extinction process^57^.

As shown by our data, the development of euxinic conditions at the LOMEI-2 was not related to volcanism (Fig. 2), but to the large amount of organic matter burial resulted from increased availability of nutrients either input from exposed continental shelves or recycled from organic matter degradation^4^. However, at the LOMEI-2, high organic carbon burial mentioned above created euxinic conditions in bottom water, accumulating Hg and killing the survivors such as conodonts, *Hirnantia* fauna (cool water brachiopod fauna)^5^ after LOMEI-1.

## Conclusions

We present herein five Hg anomaly enrichments across the Ordovician–Silurian boundary, i.e., two anomalies in the middle and late Katian, three anomalies at LOMEI-1 (end-Katian), LOMEI-2 (late Hirnantian), and one spike in the late Rhuddanian of South China, respectively. All these Hg anomalies in the Late Ordovician and early Silurian were global or at least regional based on the global Hg chemostratigraphy correlation. The Hg positive anomalies in the middle–late Katian and in the late Rhuddanian were probably caused by primary volcanic loading. Our data suggest that during the mass extinction interval, the Hg positive anomalies at LOMEI-1 and LOMEI-2 have been controlled by different factors: i.e., volcanism probably caused the Hg anomaly at the LOMEI-1, and the development of strong euxinic conditions increased Hg drawdown at the LOMEI-2. Volcanism during the Middle and late Katian would probably enhance the expansion of euxinia at the end of Katian by long-term weathering of volcanic deposits, and was finally responsible for the LOMEI-1. Furthermore, there was no or weak volcanic loading of Hg during the LOMEI-2. Hg enrichment during this time was related to euxinic condition in water column, suggesting that the LOMEI-2 was linked to euxinia.
